# Complete Chloroplast Genome Sequence of Holoparasite *Cistanche deserticola* (Orobanchaceae) Reveals Gene Loss and Horizontal Gene Transfer from Its Host *Haloxylon ammodendron* (Chenopodiaceae)

**DOI:** 10.1371/journal.pone.0058747

**Published:** 2013-03-15

**Authors:** Xi Li, Ti-Cao Zhang, Qin Qiao, Zhumei Ren, Jiayuan Zhao, Takahiro Yonezawa, Masami Hasegawa, M. James C Crabbe, Jianqiang Li, Yang Zhong

**Affiliations:** 1 Ministry of Education Key Laboratory for Biodiversity Science and Ecological Engineering, School of Life Sciences, Fudan University, Shanghai, China; 2 College of Life Science and Technology, Shanxi University, Taiyuan, China; 3 Faculty of Creative Arts, Technologies and Science, Institute of Biomedical, Environmental Science and Technology, University of Bedfordshire, Luton, United Kingdom; 4 Wuhan Botanical Garden, Chinese Academy of Sciences, Wuhan, China; 5 Institute of Biodiversity Science and Geobiology, Tibet University, Lhasa, China; Wuhan University, China

## Abstract

**Background:**

The central function of chloroplasts is to carry out photosynthesis, and its gene content and structure are highly conserved across land plants. Parasitic plants, which have reduced photosynthetic ability, suffer gene losses from the chloroplast (cp) genome accompanied by the relaxation of selective constraints. Compared with the rapid rise in the number of cp genome sequences of photosynthetic organisms, there are limited data sets from parasitic plants.

**Principal Findings/Significance:**

Here we report the complete sequence of the cp genome of *Cistanche deserticola*, a holoparasitic desert species belonging to the family Orobanchaceae. The cp genome of *C. deserticola* is greatly reduced both in size (102,657 bp) and in gene content, indicating that all genes required for photosynthesis suffer from gene loss and pseudogenization, except for *psb*M. The striking difference from other holoparasitic plants is that it retains almost a full set of tRNA genes, and it has lower *dN/dS* for most genes than another close holoparasitic plant, *E. virginiana*, suggesting that *Cistanche deserticola* has undergone fewer losses, either due to a reduced level of holoparasitism, or to a recent switch to this life history. We also found that the *rpo*C2 gene was present in two copies within *C. deserticola*. Its own copy has much shortened and turned out to be a pseudogene. Another copy, which was not located in its cp genome, was a homolog of the host plant, *Haloxylon ammodendron* (Chenopodiaceae), suggesting that it was acquired from its host via a horizontal gene transfer.

## Introduction

The chloroplast is an important organelle in the plant cell, and its central function is to carry out photosynthesis and carbon fixation. In general, the chloroplast (cp) genome is highly conserved among seed plants with two copies of a large inverted repeat (IR) separated by small single copy (SSC) and large single copy (LSC) regions [1]. It usually contains 110–130 unique genes, which can be roughly divided into three large groups according to their functions: genetic system genes, photosynthesis genes and conserved open reading frames with miscellaneous functions [2].

However, a small group of angiosperm plants appear to have escaped from this dominant pattern by evolving the capacity to gain the water, carbon and nutrients via the vascular tissue of the parasitized host’s roots or shoots. This means that these parasitic plants have reduced (or no) photosynthetic ability, and no longer need genes that encode photosynthetic proteins. With the selective constraints on their cp coding genes relaxed, gene losses occur in these parasitic plants [3]. It is estimated that approximately 1% of all angiosperm species have resorted to a parasitic lifestyle, which has independently evolved 12 or 13 times [4]. Compared with a rapid rise in the number of cp genomes of photosynthetic organisms available on NCBI (254 in Viridiplantae, as of December 4, 2012), there are limited data sets from parasitic plants, especially from the completely non-photosynthetic species. In higher plants, the cp genome of holoparasite *Epifagus virginiana* in the family Orobanchaceae was sequenced first [5], followed by four species from the holoparasitic genus *Cuscuta* [6], [7], and three mycoheterotrophic plants, including *Aneura mirabilis* [8], *Rhizanthella gardneri* [9], and *Neottia nidus-avis* [10]. However, only one species of the completely non-photosynthetic plants, *E. virginiana*, which exploit other plants via direct connections rather than by mycorrhizal fungi, has been comprehensively analyzed in its cp genome structure and composition.

Orobanchaceae, as taxonomically redefined by a series of recent molecular studies, comprise around 89 genera and more than 2,000 species, making it the largest predominantly parasitic angiosperm family, the majority of which are facultative or obligate root parasites [11]–[16]. It contains all levels of parasitic ability ranging from nonparasitic to hemiparasitic and holoparasitic [12], [17]. Therefore, analyses of cp genomes of other holoparasitic species within the family Orobanchaceae could confirm the common attributes of non-photosynthetic evolution and provide point for genetic analysis of cp genome evolution.

In Orobanchaceae, *Cistanche* is a worldwide genus of holoparasitic desert plants. Specifically, *C. deserticola*, commonly known as desert-broomrape and traditionally used as an important tonic in China and Japan, is distributed in Northwest China and the Mongolian People’s Republic, and is also considered to be an endangered wild species in recent years due to increased consumption by humans [16]. *C. deserticola* is parasitized on the roots of psammophyte *Haloxylon ammodendron* (Chenopodiaceae), which mainly inhabit deserts and semi-deserts due to its high tolerance to drought and salinity. Similar to *E. virginiana*, *C. deserticola* is a completely non-photosynthetic species and usually grows underground. A number of studies about the chemical components or pharmacological effects of this species have been reported [1], [18], [19]. Further analysis of its cp genome structure and composition could provide new insights on the evolution of the parasitic cp genome.

Attributed to the direct connections between parasitic plants and their hosts, which allows the channelling of metabolites, such as sugars, amino acids and perhaps nucleic acids in the form of mRNA, direct haustorial contact between them usually facilitates a horizontal gene transfer (HGT) from a donor to a recipient plant [20]. HGT, known as exchange of genes across mating barriers, has played a major role in bacterial evolution. In recent years, increasing studies have reported HGT being recognized as a significant force in the evolution of eukaryotic genomes [21], [22]. In plants, the evolutionarily earliest examples of HGT might be the endosymbioses that gave rise to mitochondria and chloroplasts [23], [24]. Since the emergence of HGT events, usually detected as incongruences in molecular phylogenetic trees, a considerable number of studies have suggested gene exchanges between hosts and parasites [4], [25]–[30].

Although the HGT involving parasitic plants appears to have occurred in many parasitic lineages, the majority of reported cases of HGT have been limited to exchanges between mitochondrial genes among related species [4], [25]. Cases of HGT involving cp genomes are rare [29]. The disparity in frequency of plant-to-plant HGT between the mitochondrial and the cp genomes is considered due to an active homologous recombination system [31], [32]. It is reported that a chloroplast region including *rps*2, *trn*L-F, and *rbc*L among a group of nonphotosynthetic flowering plants, *Phelipanche* and *Orobanche* species, both from the family Orobanchaceae, were detected according to the phylogenetic trees based on available data [29].

In order to examine the effect of its non-photosynthetic life history on cp genome content, we sequenced the entire cp genome of *C. deserticola*. As a completely non-photosynthetic species from Orobanchaceae, it shows the same pattern in the process of gene loss as in chloroplasts of *E. virginiana* and other parasitic plants. We also found that *C. deserticola* has two copies of a cp gene *rpo*C2, one becoming a pseudogene, the other being horizontally acquired from the host *H. ammodendron,* according to a homology search and phylogenetic analysis.

## Materials and Methods

### Genome Sequencing and Assembly

The spikes of C. deserticola were collected from a plant base in Bayannur City of Inner-Mongolia area which was introduced from natural populations located in desert area of Inner Mongolia in northeastern China. The collecting permit was obtained from the owner (Jun Wei) of the plant base. The voucher specimen was deposited in the MOE Key Laboratory for Biodiversity Science and Ecological Engineering at Fudan University. For cp genome sequencing, total genomic DNA extraction was performed using the Plant Genomic DNA Kit (Tiangen Biotech Co., China), following the manufacturer’s instructions. The fragments of cp DNA were amplified by the polymerase chain reaction (PCR). In brief, due to loss and pseudogenizations in the cp genome of *C. deserticola*, PCR primers were designed using the reported PCR primers from several sources. The primers of the LSC region were designed using the reported conserved cp DNA primer pairs, which including 38 primer pairs as well as eight primer pairs flanking cpDNA microsatellites tested on 20 plant species from 13 families [33]. Only 14 of the 38 primer pairs are useable in *C. deserticola*. Then the primers for other regions were designed according to the primers of the cp genome available in the cp genome database [34]. Some primers were also developed from the cp genome sequences of related species (*Olea europaea* and *E. virginiana*) for specific regions. In order to amplify longer fragments, some of these primers were used combined, and some of them were designed based on the newly determined sequences of adjacent regions. By using all above primers, we covered the entire cp genome of *C. deserticola* with PCR fragments ranging in size from 500 bp to 3 kb. The overlapping regions of each pair of adjacent PCR fragments exceeded 150 bp. The amplified product was purified, and ligated into TaKaRa pMD19-T plasmids (TaKaRa BioInc, Shiga, Japan), which were then cloned into *Escherichia coli* strain DH5a. Multiple (≥6) clones were randomly selected and followed by automated sequencing using ABI 3730xl DNA Analyzer (Applied Biosystems, Foster City, CA). All fragments were sequenced 2–10 times (6-fold coverage of the *C. deserticola* cp genome on average). All these individual sequences were excluded vector, primer and low-quality reads, and then assembled using Sequencher 3.0 software (Gene Codes Corporation, USA). The inverted repeat regions (IRs) of the cpDNA were not amplified separately, but primers were designed to amplify the regions spanning the junctions of LSC/IRA, LSC/IRB, SSC/IRA and SSC/IRB. Considering two IRs cannot be distinguished by automated assembly software, we input the reads as two groups and obtained two large contigs, with each contig including one IR and its adjacent partial LSC and SSC regions. Then, the two large contigs were manually assembled into the complete circular genome sequence.

### Genome Annotation and Molecular Evolutionary Analyses

Initial gene annotations were performed using the chloroplast annotation package DOGMA (http://phylocluster.biosci.utexas.edu/dogma/) [35]. Genes that were undetected by DOGMA, such as *psb*B, *psb*K, *trn*G-GCC, *rpo*C2, *atp*B, *acc*D, and *ycf*1, were identified by Blastn (http://blast.ncbi.nlm.nih.gov/Blast.cgi). The correctness of the annotation for all genes was additionally verified by a similarity search against the available plant cp genome sequences. The regions with similarity to known protein coding genes but lacking intact open reading frames (ORF) were identified as pseudogenes. tRNA genes were annotated using DOGMA and ARAGORN v1.2 (http://130.235.46.10/ARAGORN/) [36], and then confirmed by ERPIN (http://tagc.univ-mrs.fr/erpin/) [37]. The circular gene map of the *C. deserticola* cp genome was drawn by GenomeVx [38] followed by manual modification. An assembled and corrected sequence of *C. deserticola* cp genome was deposited in GenBank.

To estimate the selection constraint on the genes remaining in *C. deserticola* cp genome, the protein-coding genes that shared between *C. deserticola*, *E. virginiana*, and related photosynthetic species *O. europaea* were chosen to calculate the ratio of the rates of nonsynonymous and synonymous changes (*dN/dS*). *Nicotiana tabacum* was also included in the analyses to calculate *dN/dS* for photosynthetic plants. Alignment was performed using ClustalW [39]. The pairwise *dS, dN* and *dN/dS* ratios were calculated using DnaSP ver. 5 [40].

### Isolation and Sequencing of Potential HGT Gene (*rpoC2*)

In this study, we found that *C. deserticola* harbours another copy of *rpo*C2 outwith its own, which corresponds to the phylogenetic position of the host *H. ammodendron*. We propose that this copy arose via horizontal gene transfer. In order to confirm if HGT occurred across the range of C. deserticola, we sampled 50 accessions from the same plant base in Bayannur City which were introduced from five natural populations located in Alxa Left Banner, Alxa Right banner (two populations), and Urad Rear Banner in the Inner-Mongolia area, as well as Hetian in the Xinjiang area. In order to confirm if the HGT occurred across the range of *C. deserticola*, total DNA extractions from these materials and cp *rpo*C2 genes amplified by standard PCR were performed in two different labs, thus, eliminating laboratory contamination. The *rpo*C2 gene was amplified using primers f4 (5′-GATAGACATCGGTACTCCAGTGC-3′) and r6 (3′-TCATTATGGGAATGTACACGCG-5′) with the following conditions: 94°C for 2.5 min; 35 cycles each at 94°C for 1 min,55°C for 30 s, 72°C for 1 min. In *H. ammodendron*, the five clones of *rpo*C2 gene were also checked.

### Phylogenetic Analyses of the Potential HGT Gene, *rpoC2*

All the copies of *rpo*C2 sequences detected in *C. deserticola* and *H. ammodendron* were used as queries for BLASTN searches against the NCBI database (E-value <10^−3^) [41] to identify and retrieve their homologs. On the basis of Angiosperm Phylogeny Group III [42], sampling for the present study focused on members of the clade Lamiales and Caryophyllales that includes two families Orobanchaceae and Chenopodiaceae. Finally, 29 sequences were sampled for the *rpo*C2 phylogenetic analyses, using *Oryza nivara* (NC_005973) as outgroups. Sequences were unambiguously aligned manually in BioEdit 7.0.4.1 [43].

Phylogenetic analyses were performed using maximum likelihood in PAUP v. 4.0b10 [44] and Bayesian inference in MrBayes v. 3.1 [45]. The appropriate ML model of nucleotide substitution (GTR+I+G) was determined by Modeltest 3.7 [46] according to the Akaike information criteria (AIC) [47]. Relative clade support was estimated by ML bootstrap analysis of 100 replicates of heuristic searches with settings as above. Bayesian analysis was performed with MrBayes 3.1 using same model (GTR+I+G) suggested by MrModeltest v2.2 [48]. The settings for the Metropolis-coupled Markov chain Monte Carlo process were: three runs with four chains each were run simultaneously for 1*10^7^ generations, which were logged every 1000 generations. Convergence was considered to have been reached when the variance of split frequencies was <0.01. The first 2500 generations were discarded as the transient burn-in period. The 50%-majority-rule consensus of trees sampled in the Bayesian phylogenetic analysis was used to construct a phylogram.

## Results

### The cp Genome Structure of *C. deserticola*

**Figure 1:**
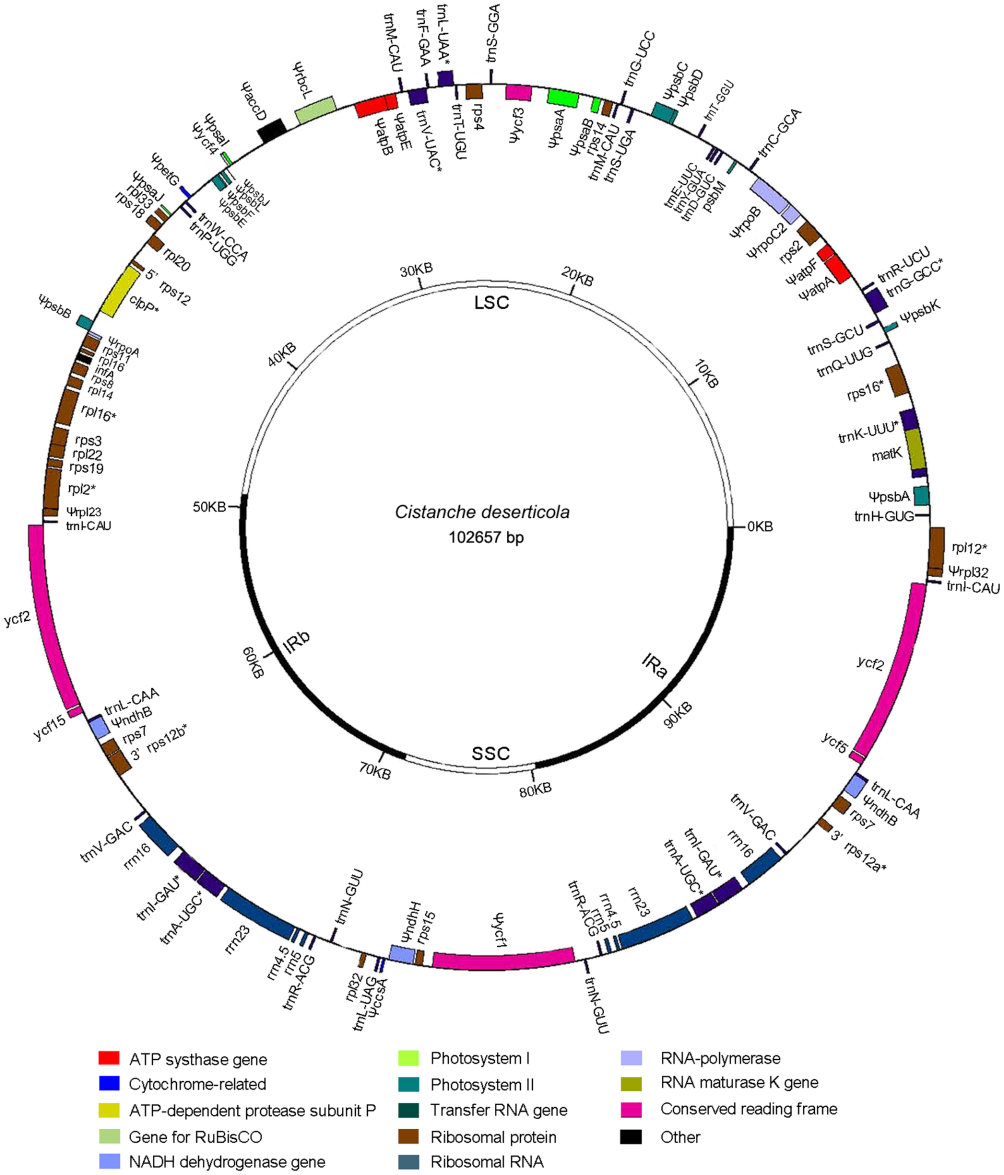
Gene Maps of the plastid chromosomes of *C. deserticola*. Genes shown inside the circle are transcribed clockwise, those outside the circle are transcribed counterclockwise. The large single copy region (LSC) and the small single copy region (SSC) are separated by two inverted repeats (IRa and IRb). Asterisks indicate intron containing genes. Pseudogenes are marked by Ψ.

As expected, the cp genome of *C. deserticola* [GenBank number: KC128846] is greatly reduced in size (102,657 bp) and in gene content. It is a quadripartite structure typical of the majority of land plant chloroplast chromosomes with a large single copy (LSC) region of 49,130 bp separated from 8,819 bp small single copy (SSC) region by two inverted repeats (IRs), each of 22,354 bp (Fig. 1). In angiosperms, it is the fourth completely nonphotosynthetic species and the eighth parasitic species of which complete sequences of the cp genome are now available. Among these species, the cp genome of *C. deserticola* is larger than those of other five holoparasites species (*E. virginiana, R. gardneri*, *N. nidus-avis*, *Cuscuta obtusiflora* and *C. gronovii*), but is smaller than those of other hemiparasitic *Cuscuta* species, which has more or less green color distributed throughout the stems and inflorescences (Tables 1, 2).

**Table 1:** Gene contents of plastidome of *Cistanche deserticola* compared to *Nicotiana tabacum* and other parasitic plants (based on this research and previous reports [6], [9], [67]).

Photosynthesis and energyproduction genes											RibosomalRNA genes									TransferRNA genes									
	Nt	Cr	Co	Cg	Ce	Cd	Ev	Rg	Nn		Nt	Cr	Co	Cg	Ce	Cd	Ev	Rg	Nn		Nt	Cr	Co	Cg	Ce	Cd	Ev	Rg	Nn
atpA	•	•	•	•	•	Ψ	Ψ	•	Ψ	rrn16	•	•	•	•	•	•	•	•	•	trnA-ugc	•	•	○	Ψ	•	•	Ψ	○	•
atpB	•	•	•	•	•	Ψ	Ψ	○	Ψ	rrn23	•	•	•	•	•	•	•	•	•	trnC-gca	•	•	•	•	•	•	Ψ	•	•
atpE	•	•	•	•	•	Ψ	○	○	Ψ	rrn4.5	•	•	•	•	•	•	•	•	•	trnD-guc	•	•	•	•	•	•	•	•	•
atpF	•	•	•	•	•	Ψ	○	○	○	rrn5	•	•	•	•	•	•	•	•	•	trnE-uuc	•	•	•	•	•	•	•	•	•
atpH	•	•	•	•	•	○	○	○	○											trnF-gaa	•	•	•	•	•	•	•	•	•
atpI	•	•	•	•	•	○	○	○	Ψ											trnfM-cau	•	•	•	•	•	•	•	•	•
ndhA	•	○	○	○	○	○	○	○	Ψ											trnG-gcc	•	•	•	•	•	•	○	○	•
ndhB	•	Ψ	○	○	Ψ	Ψ	Ψ	○	Ψ		RNA polymerase and intron maturase genes									trnG-ucc	•	•	○	Ψ	•	•	○	○	•
ndhC	•	○	○	○	○	○	○	○	Ψ											trnH-gug	•	•	•	•	•	•	•	○	•
ndhD	•	○	○	○	Ψ	○	○	○	○		Nt	Cr	Co	Cg	Ce	Cd	Ev	Rg	Nn	trnI-cau	•	•	•	•	•	•	•	•	•
ndhE	•	○	○	○	○	○	○	○	○											trnI-gau	•	•	○	Ψ	•	•	Ψ	○	○
ndhF	•	○	○	○	○	○	○	○	○	matK	•	•	○	○	•	•	•	○	Ψ	trnK-uuu	•	○	○	○	○	•	○	○	•
ndhG	•	○	○	○	○	○	○	○	○	rpoA	•	•	Ψ	○	•	Ψ	Ψ	○	○	trnL-caa	•	•	•	•	•	•	•	○	•
ndhH	•	○	○	○	○	Ψ	○	○	Ψ	rpoB	•	•	○	○	•	Ψ	○	○	Ψ	trnL-uaa	•	•	•	•	•	•	○	Ψ	•
ndhI	•	○	○	○	○	○	○	○	○	rpoC1	•	•	○	○	•	○	○	○	○	trnL-uag	•	•	•	•	•	•	•	○	•
ndhJ	•	○	○	○	○	○	○	○	Ψ	rpoC2	•	•	○	○	•	Ψ	○	○	Ψ	trnM-cau	•	•	•	•	•	•	•	○	•
ndhK	•	○	○	○	○	○	○	○	○											trnN-guu	•	•	•	•	•	•	•	○	•
petA	•	•	•	•	•	○	○	○	Ψ											trnP-ugg	•	•	•	•	•	•	•	○	Ψ
petB	•	•	•	•	•	○	○	○	○		Ribosomal protein and initiation factor genes									trnQ-uug	•	•	•	•	•	•	•	•	•
petD	•	•	•	•	•	○	○	○	○											trnR-acg	•	•	○	Ψ	•	•	•	○	•
petG	•	•	•	•	•	Ψ	○	○	Ψ		Nt	Cr	Co	Cg	Ce	Cd	Ev	Rg	Nn	trnR-ucu	•	•	•	•	•	•	Ψ	○	•
petL	•	•	•	•	•	○	○	○	Ψ	infA	Ψ	○	○	○	Ψ	•	•	•	•	trnS-gcu	•	•	•	•	•	•	•	○	•
petN	•	•	•	•	•	○	○	○	○	rpl14	•	•	•	•	•	•	Ψ	•	•	trnS-gga	•	•	•	•	•	•	Ψ	○	•
psaA	•	•	•	•	•	Ψ	○	○	Ψ	rpl16	•	•	•	•	•	•	•	•	•	trnS-uga	•	•	•	•	•	•	•	○	•
psaB	•	•	•	•	•	Ψ	○	Ψ	Ψ	rpl2	•	•	•	•	•	•	•	•	•	trnT-ggu	•	•	•	•	•	•	○	○	•
psaC	•	•	•	•	•	○	○	○	○	rpl20	•	•	•	•	•	•	•	•	•	trnT-ugu	•	•	•	•	•	•	○	○	•
psaI	•	•	○	○	•	○	○	○	○	rpl22	•	•	•	•	•	•	○	○	•	trnV-gac	•	•	•	•	•	•	○	○	•
psaJ	•	•	•	•	•	○	○	○	Ψ	rpl23	•	Ψ	○	○	Ψ	Ψ	Ψ	•	•	trnV-uac	•	•	○	○	•	•	○	○	•
psbA	•	•	•	•	•	Ψ	Ψ	○	○	rpl32	•	•	○	○	•	•	○	○	•	trnW-cca	•	•	•	•	•	•	•	•	•
psbB	•	•	•	•	•	Ψ	Ψ	○	○	rpl33	•	•	•	•	•	•	•	Ψ	•	trnY-gua	•	•	•	•	•	•	•	•	•
psbC	•	•	•	•	•	Ψ	○	○	Ψ	rpl36	•	•	•	•	•	•	•	•	•										
psbD	•	•	•	•	•	Ψ	○	○	Ψ	rps11	•	•	•	•	•	•	•	•	•		Other essential genes								
psbE	•	•	•	•	•	○	○	○	○	rps12	•	•	•	•	•	•	•	Ψ	•										
psbF	•	•	•	•	•	Ψ	○	○	○	rps14	•	•	•	•	•	•	•	•	•		Nt	Cr	Co	Cg	Ce	Cd	Ev	Rg	Nn
psbH	•	•	•	•	•	○	○	○	○	rps15	•	•	•	•	•	•	○	○	•	ClpP	•	•	•	•	•	•	•	•	•
psbI	•	•	•	•	•	○	○	○	○	rps16	•	Ψ	○	○	Ψ	•	○	○	Ψ	accD	•	•	•	•	•	Ψ	•	•	•
psbJ	•	•	•	•	•	Ψ	○	○	○	rps18	•	•	•	•	•	•	•	•	Ψ	cemA	•	•	•	•	•	○	○	○	○
psbK	•	•	•	•	•	Ψ	○	○	Ψ	rps19	•	•	•	•	•	•	•	•	•	ccsA	○	•	•	•	•	Ψ	○	○	○
psbL	•	•	•	•	•	Ψ	○	○	○	rps2	•	•	•	•	•	•	•	•	•	ycf1	•	•	•	•	•	Ψ	•	•	•
psbM	•	•	•	•	•	•	○	○	Ψ	rps3	•	•	•	•	•	•	•	•	•	ycf2	•	•	•	•	•	•	•	•	•
psbN	•	•	•	•	•	○	○	○	○	rps4	•	•	•	•	•	•	•	•	•	ycf3	•	•	•	•	•	Ψ	○	○	Ψ
psbT	•	•	•	•	•	○	○	○	○	rps7	•	•	•	•	•	•	•	•	•	ycf4	•	•	•	•	•	Ψ	○	○	○
rbcL	•	•	•	•	•	Ψ	Ψ	○	Ψ	rps8	•	•	•	•	•	•	•	•	•	ycf15	•	Ψ	○	Ψ	Ψ	•	Ψ	○	○

Nt, *Nicotiana tabacum*; Cr, *Cuscuta reflexa*; Co, *Cuscuta obtusiflora*; Cg, *Cuscuta gronovii*; Ce, *Cuscuta exaltata*; Cd, *Cistanche deserticola*; Ev, *Epifagus virginiana*; Rg, *Rhizanthella gardneri*; Nn, *Neottia nidus-avis*; Ψ, pseudogene; •, present; ○, missing.

**Table 2:** Global features of *Cistanche deserticola* plastidome compared to *Nicotiana tabacum* and other parasitic plants.

Species	Accession no	Size (nt)	Numberof genes^a^	Number of pseudogenes	Number of tRNAs^b^	number of genes with introns	Reference
*Rhizanthella gardneri*	GQ413967	59,190	37	6	9	4	[9]
*Epifagus virginiana*	NC_001568	70,028	53	18	17	4	[5]
*Cuscuta obtusiflora*	NC_009949	85,280	98	1	24	5	[7]
*Cuscuta gronovii*	AM711639	86,744	99	5	24	5	[6]
*Neottia nidus-avis*	JF325876	92,060	74	28	28	8	[10]
*Cistanche deserticola*	KC128846	102,657	77	32	30	14	this study
*Cuscuta reflexa*	AM711640	121,521	115	4	29	16	[6]
*Cuscuta exaltata*	NC_009963	125,373	112	8	29	16	[7]
*Nicotiana tabacum*	NC_001879	155,939	151	1	30	23	[68]

Data in this table came from analyses using sequences on GenBank or plastid chromosomes gene maps in their original report.

a Number of genes excluding the pseudogenes. Duplicated genes in the IR regions were counted twice.

b Duplicated tRNAs were counted both.

When the IR is considered only once, the cp genome of *C. deserticola* contains 60 genes, encoding 27 proteins, 4 ribosomal RNAs (rRNA) and 30 transfer RNAs (tRNA). The positions of 61 genes, including 29 unique and 16 duplicated ones in the IRs regions, were localized on the map (Fig. 1). The cp genome of *C. deserticola* has an overall GC content of 36.8%, which is similar to *E. virginiana* (36%) but slightly lower than the photosynthetic species *Nicotiana tabacum* (37.8%). Like other land plants [49], [50], GC content is unevenly distributed across the *C. deserticola* cp genome. The highest GC content is in the IRs (43%), reflecting the high GC content of rRNA genes, and the lowest is in the SSC (27.5%) region.

Although *C. deserticola* has a relatively larger cp genome sequence, it also exhibited severe physiological reductions: all genes required for photosynthesis (encoding photosystem I and II components, cytochrome b6f complex, NAD(P)H dehydrogenase, photosystem assembly factors (*ycf*3, *ycf*4) and ATP synthase) suffered gene losses and pseudogenizations except for *psb*M. Additional pseudogenization is also seen in genes encoding cp-encoded RNA polymerase (*rpo*), Cytochrome c biogenesis protein (*ccs*A), and Acetyl-CoA carboxylase (*acc*D). *C. deserticola* retains many genes of the translation machinery, including 8 *rpl* genes, 11 *rps* genes, and an initiation factor, infA. Only *rpl*23 is apparently a pseudogene with nonfunctional reading frames (Table 1).

In *E. virginiana*, a total of five tRNAs were pseudogenized and eight tRNAs were lost [51]. In contrast, *C. deserticola* retains almost all the rRNA and tRNA genes: two identical copies of rRNA gene clusters (16S-23S-4.5S-5S) were found in the IR regions; 30 different tRNAs, which can recognize all 61 codons present in the cp genes, were identified (Table 1, Fig. 1). Intron content of genes retained in the *C. deserticola* cp genome is conserved with other seed plants: it has 11 genes with introns, six in tRNAs and 5 in protein coding genes. Two of the 12 intron-containing genes have a single intron and two genes, *clp*P and *rps*12, have two introns. All of these belong to the group II intron, whereas *trn*L-UAA is the only group I intron. Among the tRNA genes, *trn*K-UUU has a special role, since the only RNA maturase gene (*mat*K) found on the cp genome was located in its intron [52]. Unlike other parasitic plants, *C. deserticola* harbours the complete *trn*K-UUU gene, including its intron *mat*K gene (Table 1, Fig. 1).

**Figure 2:**
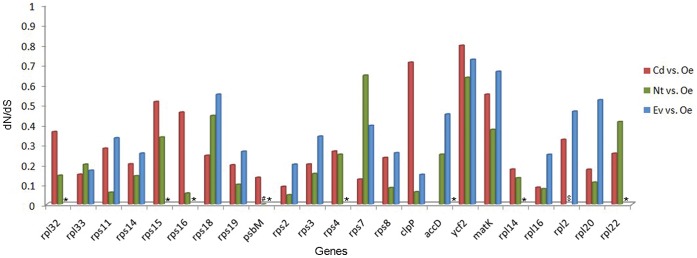
Pairwise dN/dS value of *C. deserticola* (Cd), *E. virginiana* (Ev) and *N. tabacum* (Nt) vs. *O. europaea* (Oe) for all shared protein-coding genes. *indicates gene lost, ^#^indicates the absence of non synonymous substitutions, ^$^indicates the absence of synonymous substitutions.

The examination of pairwise *dN/dS* ratio for the alignable genes shared between *C. deserticola*, *E. virginiana* and their autotrophic relatives demonstrates that most of genes are under greater constraint in fully nonphotosynthetic *E. virginiana* and *C. deserticola* than photosynthetic species *O. europaea* and *N. tabacum* (Fig. 2). In addition, of 15 protein-coding genes shared between *E.virginiana* and *C. deserticola*, 13 genes have a higher *dN/dS* in *E.virginiana* than in *C. deserticola* (Fig. 2).

### Horizontal Transfer of *rpo*C2 from Host to Parasitic Plants

**Figure 3:**
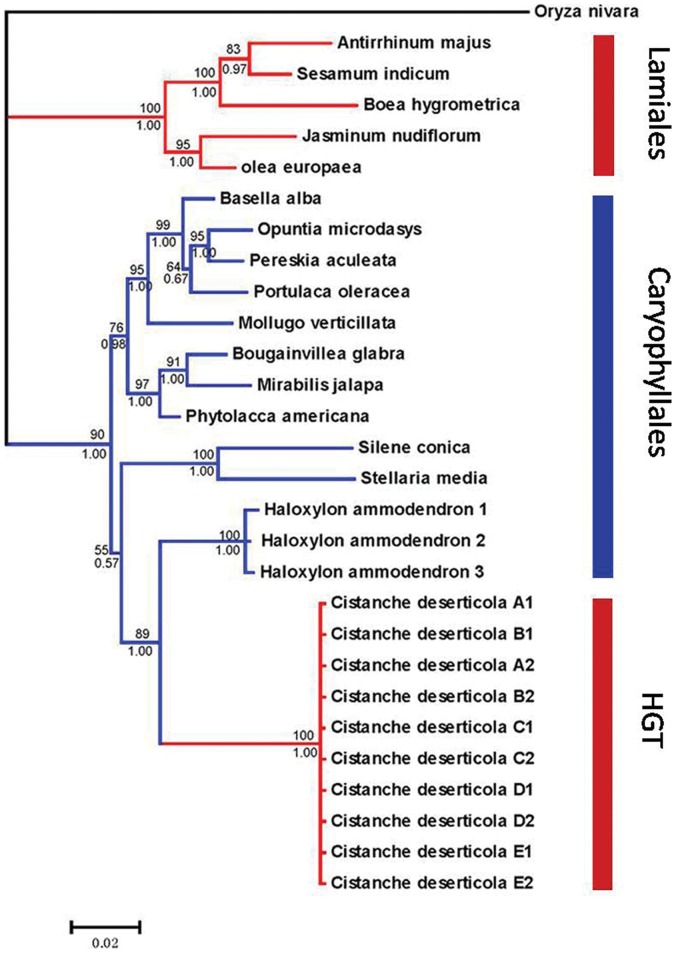
Phylogenetic evidence for horizontal gene transfer of the plastid rpoC2 from *Haloxylon ammodendron* to *Cistanche deserticola.* Lamiales are coloured in red, and Caryophyllales are coloured in blue. While the ten *C. deserticola* sequences involved in horizontal gene transfer are coloured in red. Numbers at nodes are posterior probabilities >0.60 and maximum likelihood bootstrap values >60. The Genebank number: *Oryza nivara*, NC_005973; *Antirrhinum indicum*, GQ997028; *Sesamum indicum*, NC_016433; *Boea hygrometrica*, NC_016468; *Jasminum nudiflorum*, NC_008407; O*lea europaea*, NC_013707; *Basella alba*, HQ843359; *Opuntia microdasys*, HQ843375; *Pereskia aculeata*, HQ843376; *Portulaca oleracea*, HQ843380; Mollugo verticillata, HQ843373; *Bougainvillea glabra*, HQ843360; *Mirabilis jalapa*, HQ843372; *Phytolacca americana*, HQ843378; *Celosia cristata*, HQ843361; *Spinacia oleracea*, NC_002202; *Silene conica*, NC_016729; *Stellaria media*, HQ843386; *Cistanche deserticola* (HGT), KC543998.

**Figure 4:**
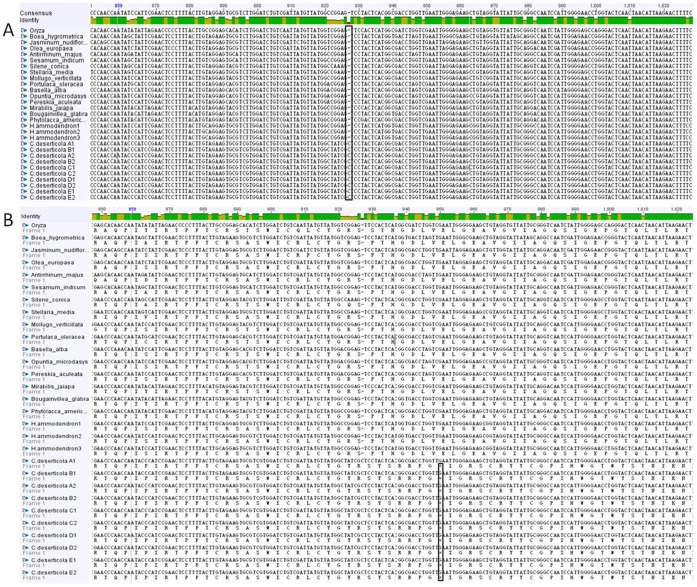
Position of inserted cytosine within the transferred rpoC2 gene. (A) Alignment of the nucleotide sequences of transferred rpoC2 gene amplified from parasite and host with intact open reading frames of other related species. The inserted cytosine was labeled with colored vertical lines. (B) Inserted cytosine resulted in followed premature termination codon in the transferred rpoC2 in *Cistanche deserticola*.

We used the general primers of *rpo*C2 to amplify the total DNA of *C. deserticola* and subsequent cloning. Contrary to our expectation, the sequences obtained resemble the genes of *H. ammodendron* but not *C. deserticola*, based on sequence similarity through BLAST and phylogenetic trees (GenBank number: KC543998, Fig. 3). This raised the possibility that HGT may have occurred between the parasite and its host. In order to confirm this result, we ruled out that the results were due to contamination or mixing-up of templates by repeating the experiment in a different laboratory. The results of the amplification are congruent with the previous results. Then, we confirmed the presence of the *H. ammodendron* type copy in 46 accessions out of 50 samples from five *C. deserticola* populations by using the same specific primers. Four of the accessions’ lack of amplification was probably due to poor DNA quality. The transferred *rpo*C2 copy (*H. ammodendron* type) amplified from *C. deserticola* is about 1050 bp, and covers amino acid positions 98–443 (nucleotide positions 294–1329) of the *rpo*C2 gene of *O. europea*. To clarify the evolutionary characteristics of the *rpo*C2 fragment transferred from *H. ammodendron* to *C. deserticola*, we aligned the nucleotide sequences of *H. ammodendron* type *rpo*C2 amplified from both of two plants with intact open reading frames of other related species. The results indicated that the transferred *rpo*C2 fragment differed from functional copies in a few point mutations and one key nucleotide insertion (C in 927 bp), which resulted in several subsequent premature termination codons and frame shifts mutations (Fig. 4). Because the *H. ammodendron* type *rpo*C2 was not found in the complete cp genome of *C. deserticola*, we speculated it should be transferred into the nuclear or mitochondrial genome.

However, *C. deserticola*’s own *rpo*C2 copies were not detected by PCR amplification using specific primers, which make us consider that this gene was lost or turned out to be a pseudogene. Thus, we searched the finished cp genome of *C. deserticola* with *rpo*C2 homologues by the BLAST method. The results shown that *C. deserticola* also retains its own significant shortened *rpo*C2, which has turned out to be a pseudogene of only 439 bp.

### Phylogenetic Analysis of Transferred *rpo*C2 Gene

The HGT result was further supported by our phylogenetic analysis. Maximum likelihood and Bayesian trees constructed using the two methods described earlier gave congruent results (Fig. 3). The two orders Lamiales and Caryophyllales confirmed as well as supported distinct clades in the phylogenetic tree. The transferred *rpo*C2 is located in the clade Caryophyllales (host clade) but does not cluster inside Lamiales (parasitic clade), which forms a clade with a relatively strong bootstrap support. The retained *rpo*C2 (*C. deserticola* type copy) was not used in this analysis because its sequence was severely fragmented when align with other homologs. The sequence alignment and the phylogenetic distribution of the *rpo*C2 in Chenopodiaceae suggest that the horizontal gene transfer happened between the host *H. ammodendron* and parasitic plant *C. deserticola*.

## Discussion

### Gene Losse in the cp Genome of *C. deserticola*

Compared to more than 250 completely sequenced cp genomes of photosynthetic plants, the number of fully sequenced cp genomes of non-photosynthetic plants is very small. To date, only eight heterotrophic species, exhibiting parasitic lifestyles and having strongly reduced cp genomes, have been thoroughly investigated with respect to their cp genome sequences [5]. In this study, we have sequenced the cp genome of *C. deserticola*, a holoparasitic species from Orobanchaceae with the expectation that comparison of cp genomic features between these two relatives will provide further insights on parasitic cp genome evolution.

The overlapping PCR products have indicated the reduced circular form of the cp chromosomes in *C. deserticola*. Similar to *E. virginiana*, almost all of its photosynthetic genes have been lost or have become pseudogenes after the loss of a major metabolic function. It is different from other heterotrophic plants in many ways: it retains almost all the tRNA genes; the photosynthetic gene *psb*M remains as residues and others suffered gene pseudogenizations rather than losses as *E. virginiana*; *C. deserticola* harbours complete *trn*K-UUU gene but not its intron *mat*K gene, and so on.

Some parasitic species exhibit extensive losses of tRNA genes (Table 1). In *E. virginiana*, a total of 13 tRNAs were pseudogenized or lost. As in photosynthetic plants, *C. deserticola* encompasses around 30 tRNA genes in cp genomes, and it is the only one of parasitic plants which possessed a full cp tRNA set as nonparasitic plants. This suggests that the loss of the transfer RNA genes from the cp genome occurred later than those of photosynthesic genes.

Most of the splicing factors are nuclear-encoded, but one maturase protein is encoded by a cp gene, *mat*K, which was located within an intron of *trn*K-UUU [52], [53]. The *trn*K gene is lost in all parasitic angiosperm cp genomes except for *C. deserticola* and *Neottia nidus-avis* (Table 1). In the *Neottia* cp genome, the intron *mat*K is a pseudogene with strong divergence of its 5′end compared to other photosynthetic orchids [10]. In contrast, in *C. reflexa*, *C. exaltata*, and *E. virginiana*, *matK* has been retained as a free-standing gene [52], [54]. Unlike other parasitic angiosperm species, neither the *trn*K-UUU gene nor its intron *mat*K gene was missing in *C. deserticola*. It has been reported that *matK* is also needed for splicing other chloroplast group II introns in the cp genome [55]. Thus the retaining of *matK* in *C. deserticola* is not surprising because its cp genome has retained 9 group IIa introns (including *rpl*2, *rpl*16, *rps*12, *clp*P, *trn*A-UGC, *trn*I-GAU, *trn*K-UUU, *trn*G-UCC, *trn*V-UAC). While the *trnK* gene exists in the *C. deserticola* cp genome, which was similar to photosynthetic plants, this may suggest the plant has undergone fewer losses, either due to a function of reduced level of holoparasitism, or a recent switch to this life history [56].

The entire set of chloroplast NAD (P) H dehydrogenase consisting of 11 genes has been lost or turned into pseudogenes without exception in *C. deserticola*. What is interesting is that a loss of *ndh* genes was also present in all sequenced cp genomes of parasitic plants investigated to date, regardless of the degree of evolutionary degradation of photosynthetic capacity (Table 1). It was confirmed that cp-encoded *ndh* genes were first lost in the transition to heterotrophy [7], [57]. It has been speculated that the condensation of the genome by loss of many non-coding regions and unimportant parts of the cp genome is an early reaction of the cp genome to the parasitic lifestyle [6].

After calculating *dN/dS* for shared cp genes between *E. virginiana, C. deserticola* and two photosynthetic species, an obvious trend of relaxed selection was revealed in both fully nonphotosynthetic species with higher *dN/dS*. It may indicate that these genes were suffering an initial stage of pseudogenization. However, *C. deserticola* has lower *dN/dS* for more genes than *E. virginiana*, which suffered a high degree of gene loss and pseudogenization, further indicating *C. deserticola* may undergo reduced level of holoparasitism or a recent switch to this life history. The gene *psb*M, which was the only one photosynthetic gene retained in *C. deserticola*, showed a higher *dN/dS* than in photosynthetic species (*dN/dS = *0), suggesting advent of relaxed selection and initial stage of pseudogenization in this gene in *C. deserticola*. However, some unexpected high *dN/dS* were also found in *rpl*33, *rps*7 and *rpl*22 in photosynthetic species. The short length of sequences may reduce the reliability of dN/dS estimation in these genes [58].

### HGT from *H. ammodendron* to *C. deserticola*

HGT in parasitic systems has been detected by using phylogenetic trees when a DNA sequence obtained from a parasite is placed closer to its host rather than with its closest relatives. Unexpectedly we had a windfall in the process of amplifying the cp genome sequence of *C. deserticola*. One of these sequences, *rpo*C2 gene, was present in two copies within this parasite and one of them was a homolog of their host and led to conflicting phylogenies. The most reasonable explanation for our results is that cp *rpo*C2 gene in *C. deserticola* was acquired from its host, *H. ammodendron* via HGT. In order to confirm the results and provide special opportunities for studying the evolutionary dynamics of HGT at the population level, we also collected 50 samples from five populations and successfully amplified transferred the *rpo*C2 gene from 46 accessions. In addition, the events present in most individuals spanning Xinjiang and Inner Mongolia, may suggest that the HGT of *rpo*C2 probably occurred in a *C. deserticola* common ancestor of these populations, which expanded into its present wide distribution quickly.

So far, the incidence of HGT in the family Orobanchaceae is high, including one nuclear HGT event which occurred between parasitic *Striga hermonthica* (Orobanchaceae) and its host *Sorghum bicolor* (Poaceae), as well as a chloroplast region including *rps*2, *trn*L-F, and *rbc*L in a group of non-photosynthetic members (*Orobanche* and *Phelipanche*) of Orobanchaceae [29], [59]. Our study shows that cp *rpo*C2 has transferred from *H. ammodendron* to *C. deserticola* via HGT. However, it is impossible to presume the localization of the transferred *rpo*C2 based on the available data. We just could rule out its location in the cp genome according to our completed cp genome of *C. deserticola*. This agrees with the reports that events of foreign DNA transferred into the cp genome are rare [60], [61]. The possibility of disparity between plant mitochondrial and nuclear genomes vs. cp genomes in rates of HGT is that the mitochondrion and nuclear genomes contain much more non-coding DNA than compact cp genomes [62], [63].

As desert plants, *H. ammodendron* and *C. deserticola* have developed an extremely specialized set of morphological, biochemical and molecular traits to adapt scare nutrients and water in the soil, such as loss of leaves and the development of haustoria in *C. deserticola*. With this feeding organ, *C. deserticola* can extract water and nutrients from the parasitized host, including the nucleic acids in the form of mRNA. It is why HGT appears to be facilitated by the direct physical association between the parasite and its host in the parasitic systems.Moreover, *C. deserticola* is a typical root parasite, meaning they are usually in contact with its host through meristems. In plants, meristems are less protected than the germlines in most multicellular animals [22]. Therefore, the genes, which transferred to the root apical meristem, could have the opportunity to be integrated in the genome and transmitted to the next generation.

In our study, either the transferred *rpo*C2 or its native copy appear to be non-functional pseudogenes in *C. deserticola*. Previous work has reported plant mtDNA pseudogenes that are transcribed and edited, so this raises the possibility that some of these genes may actually be functional [64], [65]. The fact is that acquiring a new gene can lead to an obvious benefit to living in that particular environment. *H. ammodendron,* which is distributed across dry deserts and salt pans, has high tolerance to osmotic and salt stress [66]. We postulate that *C. deserticola* could not only obtain the carbohydrate, minerals and water, but also the straightforward source of useful genetic information from the neighbour already adapted to that environment. In the ‘genomic era’, future work is still needed to discover more HGT events in this pair of host and parasite by next generation sequencing, especially genes in mitochondrial and nuclear genomes.
